# Galangin Nanoparticles Protect Acetaminophen-Induced Liver Injury: A Biochemical and Histopathological Approach

**DOI:** 10.1155/2022/4619064

**Published:** 2022-08-10

**Authors:** Arezoo Mohammadi, Sohrab Kazemi, Inas Molayousefian, Marzieh Pirzadeh, Ali Akbar Moghadamnia

**Affiliations:** ^1^Student Research Committee, Health Research Center, Babol University of Medical Sciences, Babol, Iran; ^2^Cellular and Molecular Biology Research Center, Health Research Institute, Babol University of Medical Sciences, Babol, Iran; ^3^Department of Pharmacology and Toxicology, School of Medicine, Babol University of Medical Sciences, Babol, Iran

## Abstract

One of the main causes of acute liver failure is overdose with acetaminophen. Excessive consumption of acetaminophen leads to the production of NAPQI (N-acetyl-p-benzoquinone imine) through the activity of the enzyme cytochrome c oxidase. For this purpose, the effect of galangin nanoparticles with antioxidant activities will be evaluated for the treatment of acetaminophen-induced hepatotoxicity. In this study, after the synthesis of galangin nanoparticles and particle size determination, mice were divided into six groups. Before treatment, a single dose (350 mg/kg) of acetaminophen was administered by gavage in all groups. The activity of alanine aminotransferase (ALT), aspartate aminotransferase (AST), alkaline phosphatase (ALP), as well as biochemical factors FRAP and MDA in serum were measured and a histopathological study was performed. The prepared nanoparticles produced in this research were characterized by the SEM, DLS, and ZETA potential, and the average particle size was obtained in the range of 150 nm. Serum levels of liver enzymes (AST and ALT) in the nanoparticle group decreased significantly compared with the control group (*P* < 0.05). In the group without treatment, the activity of aspartate aminotransferase (AST) and alanine aminotransferase (ALT) enzymes increased significantly compared with the treatment groups. Also, galangin nanoparticles, at a dose of 20 mg/kg, improve cell damage in hepatocytes and preserve the tissue structure of the liver. Galangin nanoparticles reduce the acetaminophen-induced hepatotoxicity by reducing the number of liver function indices. According to our findings, the liver-protective effects of the nanoparticle may be due to its antioxidant properties.

## 1. Introduction

With its analgesic and antifever properties, and its low risk at the recommended dose, acetaminophen is widely used as an over-the-counter drug [1]. Acetaminophen poisoning is one of the significant causes of liver damage, and the use of this drug in chronic form may cause serious health problems [2, 3]. Acetaminophen is converted to non-toxic metabolites in phase II metabolism. Consumption of excessive amounts of it saturates other pathways of glucuronidation and sulfation. It leads to the production of its toxic metabolite, N-acetyl-*ρ*-benzoquinone imine (NAPQI), which is electronegative and bonds to the liver proteins and it is the product of cytochrome P450 CYP2E1 [4–6]. Primary toxicity of NAPQI is due to the reaction with glutathione (GSH), NAPQI initially stores glutathione in liver cells, and then forms covalent bonds with cellular proteins. Acetaminophen-induced hepatotoxicity occurs when glutathione storage reaches 30% below the normal range [7, 8] by forming reactive oxygen species, which leads to liver cell necrosis and apoptosis [9], and when the glutathione storage is finished, the toxic metabolites of acetaminophen bond to a significant number of mitochondrial and cytosolic proteins and disrupts mitochondrial respiration. It also reduces the level of hepatocellular adenosine triphosphate [9, 10]. During this process, the mitochondrial permeability transition pore opens, and cytochrome c is released from the mitochondria. Several cytochrome P450 enzymes can convert acetaminophen to NAPQI, which includes CYP2E1, CYP1A2, CYP3A, CYP2A6, and CYP2D6. Meanwhile, CYP2E1, CYP1A2, and CYP3A play a prominent role in acetaminophen toxicity [10, 11].

Galangin (3,5,7-trihydroxyflavone) is a flavonol, a type of flavonoid. It is found in high amounts in the roots of some plants such as the *Alpinia officinarum* and has been used for various diseases in traditional Chinese medicine [12, 13]; it has antibacterial [14], anticancer [15], and antiobesity [16] properties, as well as antioxidant activity [17, 18]. Galangin stops inflammation in macrophages in conditions that are caused by lipopolysaccharides (LPS) [19], stops inflammation in the nephrotoxicity caused by cisplatin [18], and stops arthritis caused by collagen [20]. It also stops the allergic inflammation mediated by mast cells [21] and stops acute pulmonary damage caused by lipopolysaccharides (LPS) [22].

According to Susara Hewage et al., galangin significantly enhanced the expression of the glutamate-cysteine ligase catalytic subunit and increased the glutathione synthesis [23, 24] by activating the nuclear factor erythroid 2-related factor 2 (Nrf2). In a study by Qingqiong Luo et al. on mice with Con A-induced hepatitis that were pretreated with galangin, the increasing trend of several inflammatory cytokines, including IL-6 and TNF-*α* was significantly weakened [25].

The pharmaceutical compounds have low aqueous solubility and should be dissolved to be used in cells. In drug delivery systems, its solubility increases by modifying the drug molecule [26, 27]. The most commonly used method for solving this problem is the creation of particle systems. In particle systems, the active pharmaceutical ingredient is converted into particles of micro or nano sizes. These particles can protect the drug against environmental factors. The use of nanoparticles has several benefits, such as increasing the half-life of the drug in the bloodstream, improving tissue permeability, more half-life in the body, transferring poorly soluble drugs, and accumulation in the liver and spleen, and cancerous tissues [28, 29].

Since galangin stops inflammation in various diseases and the role of galangin in the treatment of acetaminophen-induced hepatotoxicity has not been studied so far, the activity of cytochrome oxidase enzymes plays a vital role in causing acetaminophen-induced hepatotoxicity. This study was conducted to investigate the possible role of galangin in reducing acetaminophen-induced hepatotoxicity.

## 2. Materials and Methods

### 2.1. Chemicals and Reagents

Galangin and silimarin were prepared by the Sigma Company (United States). DTNB (2, 20-dinitro-5, 50-dithiodibenzoic acid), TPTZ (2, 4, 6-tri (2-pyridyl)-1, 3, 5-triazine), TBA (2-thiobarbituric acid), trichloroacetic acid (TCA), tris-hydrochloric acid, glacial acetic acid, phosphoric acid, potassium chloride, ethylenediaminetetraacetic acid (EDTA), sodium acetate, ferric chloride (FeCl3_6H2O), ferrous sulfate, and hydrochloric acid were obtained from Merck (Darmstadt, Germany). All other chemicals were of analytical grade and highest purity.

### 2.2. Preparation of Galangin Nanoparticles

Nanoparticles of galangin were prepared using the deposition method [30]. For preparation of galangin nanoparticles, 20 mg galangin was added to 3 ml of ethanol solvent and was placed on a 40°C water bath until galangin was utterly dissolved in ethanol. This solution was added drop by drop to 27 ml double distilled water rotating at 14000 rpm (Ika, Germany) within five minutes until the galangin nanoparticles were prepared. The ethanol solvent was completely removed using a rotary evaporator at 40°C (Ika, Germany). A freeze dryer was used to obtain the dry powder, and the remaining solution was turned into dry powder using a freeze dryer.

### 2.3. Specifications of the Prepared Nanoparticles

Galangin nanoparticle specification was determined using a scanning electron microscope (HORIBA, Japan). The nanoparticle efficacy was determined to prepare different doses for the treatment of the mice using liquid chromatography (KNAUER, Germany) based on the following protocol: mobile phase with acetonitrile and water with a ratio of 40 : 60, current of 1 ml/min, and wavelength of 270 nm.

### 2.4. Experimental Design

Male mice weighing 25 ± 2 kg were used in this experimental laboratory study. During the experiment, the mice were kept in plastic cages with a 12/12 light cycle at room temperature. Animals were given a standard laboratory diet and water. The ethics committee approved all protocols used in this study of the Babol University of Medical Sciences (ethics committee No. IR.MUBABOL.HRI.REC.I397.039). The mice were randomly divided into six groups of six and were subject to food deprivation 24 hours before the experiment. Before treatment, five groups received a single dose of 350 mg/kg acetaminophen by gavage.

  Group 1: the healthy and control group that did not receive anything.
  Group 2: the second group received 200 *μ*l of saline that was injected intraperitoneally for ten days.
  Group 3: the third group only received a single dose of 350 mg/kg acetaminophen on the first day.
  Groups 4 and 5: after receiving a single dose of acetaminophen, the fourth and fifth group received 10 and 20 mg/kg/bw galangin nanoparticles for 10 days, respectively.
  Group 6: this group received 25 mg/kg silymarin after a single dose of acetaminophen for 10 days.

On the tenth day, the mice were anesthetized with ketamine/xylazine, and then 2^cc^ blood was collected. Then, the mice were sacrificed by guillotine, and their liver was isolated for histopathological examination. For serum separation, the blood was centrifuged for 15 minutes (3000 rpm). After separation, the serum was kept at −20°C.

### 2.5. Biochemical Analysis

The activity of alkaline phosphatase, alanine aminotransferase, and aspartate aminotransferase in serum was evaluated using commercial kits, and total antioxidant activity was evaluated by the terric-reducing ability of plasma (FRAP). Data related to liver enzymes and FRAP measurements were shown as mean ± standard deviation. In FRAP measurement, the optical absorption spectra were plotted in front of different concentrations of the standard samples. Linear equation and R2 coefficient, slope, and *y*-intercept of the calibration line were calculated.

The level of MDA was measured by the spectrophotometric method using a UV-VIS spectrophotometer (Jenway, UK) at 535 nm. MDA levels are expressed as *µ*mol/L concentrations using calibrating curves and expressed as a percentage of decrease in the MDA level compared to the acetaminophen group.

### 2.6. Histopathological Examination

Histopathological study was performed to determine liver damage. Liver tissue samples were placed in paraffin after being fixed in formaldehyde, absolute alcohol 70%, and glacial acetic acid 10%. Four- to five-micrometer sections of paraffin-embedded tissues were prepared using a microtome and were stained with hematoxylin and eosin. Finally, the stained slides were analyzed using an optical microscope (HUND-H 600 LL HP 100).

### 2.7. Statistical Analysis

The results are presented as mean ± standard deviation for three independent tests. Statistical analysis was performed for both experimental and control groups using one-way ANOVA and Tukey's post-test was used to evaluate its significance. *P* < 0.05 was considered statistically significant.

## 3. Results

### 3.1. Synthesis of Nanoparticles

**Figure 1:**
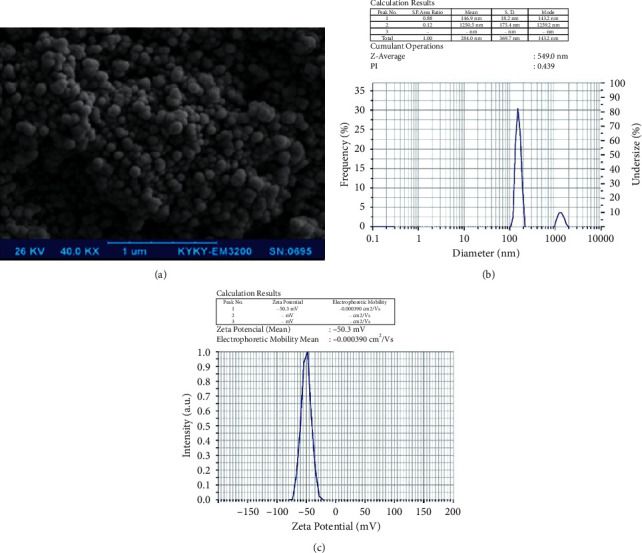
(a) SEM image of galangin nanoparticles. (b) Dynamic light scattering (DLS) of the galangin nanoparticles. (c) Zeta potential of the nanoparticles with the average amount of −50 mv.

Among the different methods of nanoparticle synthesis, the deposition method is the simplest and fastest method in which the particle size is uniformly controlled. The deposition method has advantages such as simplicity, low cost, high purity of the product, and the lack of need for organic solvents. The size of nanoparticles was shown with the SEM image in Figure 1(a). Also, the particle size of galangin nanoparticles (143.2 nm) was determined with dynamic light scattering (DLS) (Figure 1(b)). The zeta potential was found to be 50 mV (Figure 1(c)).

### 3.2. Effect of Galangin Nanoparticles on the Liver Enzyme

Liver damage was evaluated by determining the plasma levels of liver enzymes. As shown in Table 1, the plasma levels of AST, ALP, and ALT in the acetaminophen group significantly increased compared with the untreated group (*P* < 0.05); whereas, administration of galangin nanoparticle significantly reduced these markers (*P* < 0.05).

### 3.3. Effect of Galangin Nanoparticles on Biochemical Factors

**Figure 2:**
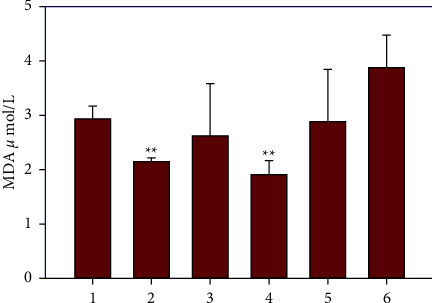
1) Control; (2) saline; (3) acetaminophen 350 mg/kg; (4) nanogalangin 10 mg/kg + acetaminophen; (5) nanogalangin 20 mg/kg + acetaminophen; (6) silymarin 25 mg/kg + acetaminophen. All values are expressed in mean ± SD, ^*∗*^*P* < 0.05, ^*∗∗*^*p* < 0.01.

**Figure 3:**
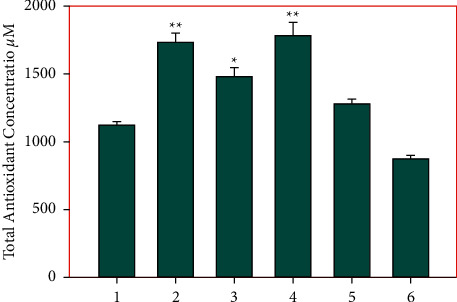
1) Control; (2) saline; (3) acetaminophen 350 mg/kg; (4) nanogalangin 10 mg/kg + acetaminophen; (5) nanogalangin 20 mg/kg + acetaminophen; (6) silymarin 25 mg/kg + acetaminophen. All values are expressed in mean ± SD, ^*∗*^*P* < 0.05, ^*∗∗*^*p* < 0.01.

In comparison with the control group, the levels of MDA in all experimental groups show that a dose-dependent decrease in the plasma MDA level is due to galangin nanoparticle treatment. The use of a high dose of galangin nanoparticles resulted in a greater significant decrease of the MDA level (Figure 2). As shown in Figure 3, the total antioxidant level (FRAP value) in the control group was significantly higher than the acetaminophen group (*P* < 0.01). The mice that received 10 and 20 mg/kg galangin nanoparticles showed increased FRAP compared to the untreated control group (*P* > 0.01). Additionally, the FRAP value increased in the groups of mice receiving 10 and 20 mg/kg galangin nanoparticles, and the positive control (silymarin) received acetaminophen compared to the untreated group receiving acetaminophen by gavage (*P* < 0.01) (Figure 3).

### 3.4. Histopathological Findings

**Figure 4:**
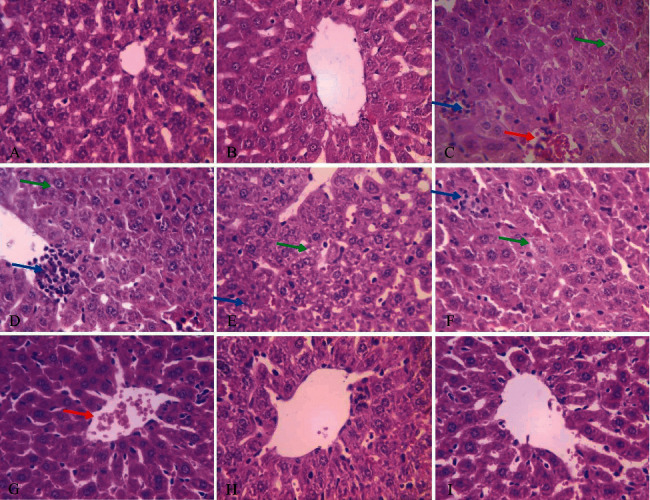
(a) Control, normal condition; (b) normal saline, normal condition; (c–e) acetaminophen, hyperemia, vascular degeneration, and infiltration; (f-g) acetaminophen + galangin 10, hyperemia, vacuolation degeneration, and infiltration inflammatory cells; H: acetaminophen + galangin 20, normal condition; I: silymarin, normal conditions.

According to the results of hematoxylin and eosin staining with 40 ×magnification (Figure 4), the pathological findings demonstrated acetaminophen-induced acute injuries of the liver tissue as hyperemia (red arrow), vacuolar degeneration (green arrow), infiltration of inflammatory cells, and increased sinusoidal space (blue arrow; images C, D, and E). The same destructive effects of acetaminophen were observed in tissue samples of the 10 mg/kg galangin nanoparticle group (image F). In contrast, hyperemia was only observed in higher concentrations such as 20 mg/kg galangin nanoparticles (image G), and normal tissue conditions were observed in the 20 mg/kg galangin group (image I), all of which suggest the positive hepatoprotective effects of galangin (similar to the positive control group (silymarin)) (Figure 4, Table 2). Furthermore, when we used 10 mg/kg galangin (image H), no destructive effect on liver tissue was observed. Also, the administration of silymarin shows the positive effects (image I).

## 4. Discussion

Today, the side effects of drugs used in the treatment of diseases of the liver necessitate further research into appropriate herbal remedies because of their ease of access, fewer side effects, less toxicity, and cheaper prices that can replace the currently used drugs. In the present study, a hepatotoxicity model was created by the administration of 350 mg/kg acetaminophen. This toxicity was specified with a significant increase in the serum levels of ALT, AST, urea, and creatinine, and the effects of various doses of galangin and other antioxidant proteins on liver damage were investigated. Acetaminophen-induced hepatotoxicity is a reliable model for the evaluation of liver-protective drugs, and several studies used various doses of acetaminophen intraperitoneally and orally for the induction of hepatotoxicity. ALT and AST are transaminase enzymes that play a crucial role in converting amino acids into keto acids. AST is predominantly present in the liver cell mitochondria, and ALT is more common in the liver cell cytoplasm; an increased level of the ALT enzyme is usually associated with an increase in AST levels. As was observed, ALT and AST levels increased significantly in mice in the acetaminophen group compared to treatment groups, which is due to increased damage of acetaminophen to liver cells, decreased cell membrane integrity, cellular leakage, and as a result, the release of these enzymes into the bloodstream.

NAPQI is an active metabolite derived from acetaminophen, which covalently bonds to lipid, protein, and DNA macromolecules. Additionally, NAPQI can react with glutathione in the liver tissue and evacuate this antioxidant compound. The results of this study showed that PCO levels in the acetaminophen group with an oral dose of 2 g/kg increased compared to group C; however, this increase was not significant. This difference may be due to the lower dose of acetaminophen used in our study. In the present study, administration of 20 mg/kg galangin nanoparticles to small white mice reduced the MDA level of the homogeneous liver tissue significantly compared to the acetaminophen group. Considering the structure of galangin nanoparticles, this compound may inhibit the protein oxidation process and thus play its antioxidant role. In a study by Heibatollah Sadeghi et al. on a mice model with liver damage induced by carbon tetrachloride, it was shown that the hydroethanolic fruit extract of *Rosa canina* decreased the levels of liver parameters (ALT, AST, and ALP) significantly compared with the control group (*P* < 0.05) [5, 31].

SOD and CAT are enzymatic antioxidants that protect tissues from lipid peroxidation. CAT prevents radical hydroxyl production and protects cellular composition from oxidative damage. Therefore, the reduction of CAT activity may lead to the accumulation of free oxygen radicals [32]. In some previous studies, it has been shown that flavonoids have anti-inflammatory properties and free radical effects [33, 34]. Total antioxidant levels were significantly lower in the acetaminophen group than in the untreated group, and in our study, the emphasis was on oxidative liver damage.

Compared to the untreated group, liver damage markers were significantly higher in the acetaminophen group. These markers show an increase of approximately 2–3 times in the acetaminophen group. In recent studies, various types of antioxidants such as quercetin, curcumin, and vitamin E and C have shown different effects in preventing the increase in the markers of gastrointestinal damage [7, 35–37]. According to the results of the present study, galangin nanoparticles and silymarin as the positive control and as antioxidants inhibit liver damage. These results emphasize the preventive antioxidant effects of galangin nanoparticles on oxidative damage caused by acetaminophen.

Our biochemical results were consistent with histological findings, while the oxidant parameters in the acetaminophen-treated group were indicative of liver damage. Centrilobular hepatic necrosis, bleeding, and sinus contractions, along with inflammatory cells in the liver were observed in histological evaluation. In our study, there was no statistically significant difference between the groups treated with silymarin and the groups treated with galangin nanoparticles used in oxidative and antioxidant parameters in liver tissues. Galangin nanoparticles and silymarin prevent liver damage from oxidative stress caused by acetaminophen almost equally. This means that galangin nanoparticles are useful as a standard acetaminophen-based antidote. Besides, according to our data, galangin nanoparticles, similar to silymarin, have no side effects on the liver.

Overall, oxidative stress is one of the leading factors in the toxicity caused by acetaminophen. Silymarin, as a well-known treatment method for acetaminophen toxicity, may be useful in all of the abovementioned cases . Galangin nanoparticles have anti-inflammatory and antioxidant effects and decrease as GSH increases. Also, some previous studies have shown that only prognostic products of CoA PA may have these effects, which indicate that the protective effect is due to this coenzyme. Except for this direct protective effect of galangin nanoparticles through CoA against oxidative damage, CoA can also help prevent cell damage by improving cellular mechanisms [34]. Galangin nanoparticles probably prevent liver damage by supporting a mitochondrial biophysical system. Given all the properties of galangin nanoparticles, this molecule is safe, affordable, and easily accessible. In the end, galangin nanoparticles can be used in cases of acute acetaminophen-induced hepatotoxicity, but more studies are required to determine its effectiveness and the method of administration in the clinical sector.

## 5. Conclusion

In summary, for the first time, this study shows that galangin nanoparticles reduce the level of hepatotoxicity induced by acetaminophen by reducing the liver function parameters (ALP, AST, and ALT). The liver-protective effects of galangin nanoparticles may be due to its antioxidant properties. Our results suggest that galangin nanoparticles may be an active alternative agent to acetaminophen-induced hepatotoxicity.

## Figures and Tables

**Table 1:** Mean and Standard Deviation of the Activity of Liver Enzymes in Serum.

Groups	Enzymes (IU/ml)		
	AST	ALP	ALT
1	77.1 ± 7.62^b^	207.44 ± 14.92^b^	101.25 ± 24.78^b^
2	139.20 ± 22.02	276.81 ± 19.69^a^	114.15 ± 20.69^a^
3	152.97 ± 19.77	363.13 ± 28.26	173.54 ± 29.47
4	110.32 ± 27.87^a^	254.15 ± 29.2^a^	117.22 ± 21.65^a^
5	92.90 ± 14.7^b^	231.50 ± 13.94^a^	101.86 ± 26.12^b^
6	86.78 ± 12.07^b^	215.38 ± 23.8^b^	102.45 ± 12^b^

Groups: (1) control; (2) saline; (3) Ac (350 mg/kg); (4) Ac + nano galangin 10 mg/kg; (5) Ac + Nano Galangin 20 mg/kg; (6) Ac + silymarin 25 mg/kg. All values are expressed in mean ± SE. ^a^*p* < 0.05. ^b^*p* < 0.01 compared to the third group.

**Table 2:** Results of Tissue Lesions in Different Groups ^a^.

Groups	Injuries		
	Hyperemia	Vacuolar degeneration	Infiltration of inflammatory cells
1	—	—	—
2	—	—	—
3	++	++	+
4	+	+	+
5	—	—	—
6	—	—	—

^a^ (+): mild; (++): medium; (+++): severe. Groups: (1) control; (2) saline; (3) Ac (350 mg/kg); (4) Ac + nano galangin 10 mg/kg; (5) Ac + nano galangin 20 mg/kg; (6) Ac + silymarin 25 mg/kg.

## Data Availability

The data used to support the study are available from the corresponding author upon request.

## Supplementary Materials

Supplementary Materials A graphical abstract is included in the supporting files (Supplementary Materials).
