# Supplementary figures and images for: Galangin Nanoparticles Protect Acetaminophen-Induced Liver Injury: A Biochemical and Histopathological Approach
Source: Evid Based Complement Alternat Med. 2022 Aug 10;2022:4619064. doi: 10.1155/2022/4619064 (PMC9385292; doi:10.1155/2022/4619064)

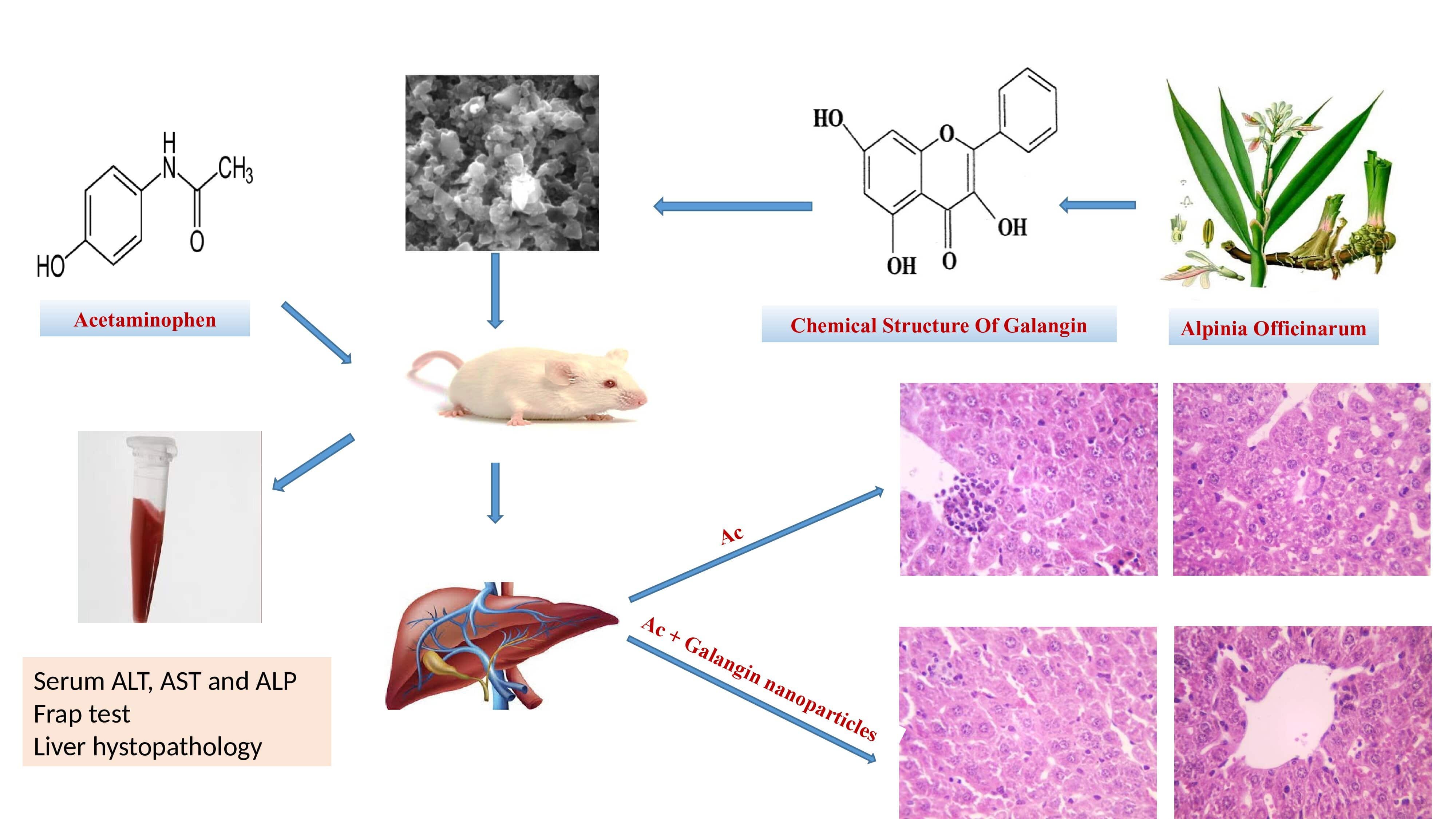

Supplement: Supplementary Materials — A graphical abstract is included in the supporting files (Supplementary Materials). [file 4619064.f1.jpg]
